# Amino acid metabolism and signalling pathways: potential targets in the control of infection and immunity

**DOI:** 10.1038/s41387-021-00164-1

**Published:** 2021-06-23

**Authors:** Daniel Tomé

**Affiliations:** UMR PNCA, AgroParisTech, INRAE, Université Paris-Saclay, Paris, France

**Keywords:** Infection, Preclinical research

## Abstract

Defences to pathogens such as SarCoV2 in mammals involves interactions between immune functions and metabolic pathways to eradicate infection while preventing hyperinflammation. Amino acid metabolic pathways represent with other antimicrobial agent potential targets for therapeutic strategies. iNOS-mediated production of NO from Arg is involved in the innate inflammatory response to pathogens and NO overproduction can induce hyperinflammation. The two Arg- and Trp-catabolising enzymes Arg1 and IDO1 reduce the hyperinflammation by an immunosuppressive effect via either Arg starvation (for Arg1) or via the immunoregulatory activity of the Trp-derived metabolites Kyn (for IDO1). In response to amino acid abundance mTOR activates the host protein translation and Coronaviruses use this machinery for their own protein synthesis and replication. In contrast GCN2, the sensor of amino acid starvation, activates pathways that restrict inflammation and viral replication. Gln depletion alters the immune response that become more suppressive, by favouring a regulatory T phenotype rather than a Th1 phenotype. Proliferating activated immune cells are highly dependent on Ser, activation and differentiation of T cells need enough Ser and dietary Ser restriction can inhibit their proliferation. Cys is strictly required for T-cell proliferation because they cannot convert Met to Cys. Restricting Met inhibits both viral RNA cap methylation and replication, and the proliferation of infected cells with an increased requirement for Met. Phe catabolism produces antimicrobial metabolites resulting in the inhibition of microbial growth and an immunosuppressive activity towards T lymphocytes.

## Introduction

Host defences to pathogen invasion, including bacteria or virus such as SarCoV2, involves in mammals a combination of initial innate immune and protective responses to eradicate infection that proceeds by directly acting on the pathogens and with locally induced inflammation, subsequently balanced by different mechanisms of immunomodulation and disease tolerance to prevent secondary harmful hyperinflammation. These processes include for a part a series of complex interactions between immune functions and metabolic pathways resulting from a convergent evolution of the two systems [1, 2]. This is particularly illustrated by the strategy exploited by higher organisms in response to pathogens infection using amino acid metabolic and signalling pathways to both restrict pathogen invasion and modulate the time-course of the immune response. Mammals control the immune response and the intracellular availability of amino acids through different mechanisms including an increase in their local catabolism and the production of amino acid-derived metabolites with both antimicrobial cytotoxic and immunomodulatory activities. Interestingly, it has been shown that these mechanisms not only result in pathogen starvation and control of infection, but also participate in regulating the potentially harmful hyperinflammatory reactivity [3]. In this context, amino acid depletion or repletion, and/or targeting different amino acid metabolites and signalling pathways can represent, in association with other antimicrobial agents, potential therapeutic strategies against microbial pathogens (Table 1).

**Table 1:** Summary of targets and strategies to control infection and immunity through amino acid metabolism and signalling pathways.

Arg	iNOS-expressing macrophages produce NO from Arg–NO overproduction induces hyperinflammation and local tissue damage: - Arg depletion using arginine-metabolising enzymes limits hyperinflammation and the availability of Arg for viral replication
	Arg1-expressing macrophages provide Pro and polyamines from Arg with immunosuppression that participate to prevent harmful hyperinflammation: - But Arg and polyamines are critical for the virus genome packaging and replication. - Pharmacological antiviral strategy targeting Arg and polyamines should favour the host while restricting Arg and polyamines for virus replication.
Trp	IDO1 causes Trp deprivation in the microenvironment and the generation of immunoactive Kyn metabolites: - Immunosuppressive regulatory effects that inhibits short-term immune response and participate to prevent harmful hyperinflammation.
Gln	Gln is essential for proliferating cells, including lymphocytes, thymocytes, and colonocytes, where it is actively used in several important metabolic processes. - Gln is the precursor for nucleotides and amino sugars. - Gln is degraded by glutaminase to Glu that is further metabolised to g-amino butyrate, glutathione, and folic acid, and is a main source of energy as precursor of intermediates components of the tricarboxylic acid cycle. - The expression of several genes in immune system cells is largely dependent on Gln availability. - Gln depletion alters the balance of the immune response that become more suppressive, by favouring a regulatory T phenotype rather than a Th1 phenotype, associated with an inhibition of IFN‑γ secretion.
Ser	Proliferating activated immune cells are dependent on Ser and activation and differentiation of T cells need enough Ser
Cys	Cys availability is critical for T-cell functions because T cells lack the enzyme converting Met to Cys.
Met	The proper methylation of RNA cap structure of SarCoV2 depends on the level of Met in the host to form SAM: - Restriction of Met availability inhibit viral replication.
Phe	Phe catabolism leads to Phe depletion and the production of H2O2 with antimicrobial toxic effects resulting in the inhibition of microbial growth and to an immunosuppressive activity towards T lymphocytes.
mTOR	Coronaviruses can exploit this cellular machinery for their own protein synthesis and replication: - Inhibition of mTOR can inhibit viral replication.
GCN2	GCN2 senses amino acid starvation and activates downstream pathways that restrict inflammation and viral replication: - Activation of GCN2 can limit hyperinflammation and reduce viral replication.

## Arginine and arginine-catabolizing enzymes iNOS and Arg1

Arginine (Arg) is a non-essential amino acid for healthy humans involved in the synthesis of proteins and in the urea cycle and acting as precursor for different molecules including glutamate (Glu), citrulline (Cit), and nitric oxide (NO), an important bioactive molecule with both cardiovascular, immunological, and neurological signalling functions, and antimicrobial cytotoxic activity [4]. In humans, Arg is supplied by the diet and by an endogenous synthesis that proceeds by two major steps, with an initial conversion in intestinal epithelial cells of dietary proline (Pro), Glu, and glutamine (Gln) to ornithine (Orn) and Cit, and the subsequent conversion of Cit to Arg both in the proximal tubules of the kidney, and in immune cells for the synthesis of NO as signalling molecule of the immune responses [5]. Arg is taken-up and transported into the cells by the solute carrier family 7 (SLC7), and the cationic amino acid transporters (CAT-1, CAT-2, and CAT-3). CAT-1 is constitutively expressed by most tissues, while CAT-2 is upregulated in murine dendritic cells (DCs) by retinoic acid, and in macrophages by Th1 and Th2-type cytokines, associated to an increase in Arg catabolizing enzymes activity and the production of Arg-derived bioactive metabolites [6, 7].

Arg catabolism involves in mammals four enzymes including nitric oxide synthase (NOS1, NOS2, NOS3), arginase (Arg1, Arg2), arginine decarboxylase (ADC), and arginine glycine amidinotransferase (AGAT) [8]. The three NOS isozymes produce NO and cit from Arg and are referred as neuronal (NOS1, or nNOS), inducible (NOS2, or iNOS), and endothelial (NOS3, or eNOS) enzymes [9]. NOS1 and NOS3 are constitutive enzymes, while NOS2 (iNOS) is induced by pro-inflammatory cytokines (including IFN-g and IL-1b) and microbial-associated products (Lipopolysaccharide, LPS). Arginase hydrolyses Arg into urea and Orn and is involved in the urea cycle and in the production from Orn, by the enzymes Ornithine decarboxylase and Ornithine amino transferase, of Glu, Pro, Cit and the bioactive polyamines (putrescine, spermidine, and spermine) involved in cell proliferation, cell repair, and modulation of inflammation. Arginase is expressed by immune cells and participates to several immune responses [10, 11]. The two isoform Arg1 and Arg2 have different tissue distribution and cellular localisation. Arg1 is a cytosolic enzyme expressed in macrophages, myeloid-derived suppressor cells (MDSCs), DCs and innate lymphoid group 2 cells in response to Th2-type cytokines (IL-4, IL-13) [10, 12, 13], and to other signalling factors associated to pathogens infection [10, 14]. In humans, Arg1 is present in the granular compartment of granulocytes in healthy subjects [15, 16], in peripheral blood mononuclear cells in subjects after injury and in activated monocytes in patients with autoimmune disease [17].

**Fig. 1:**
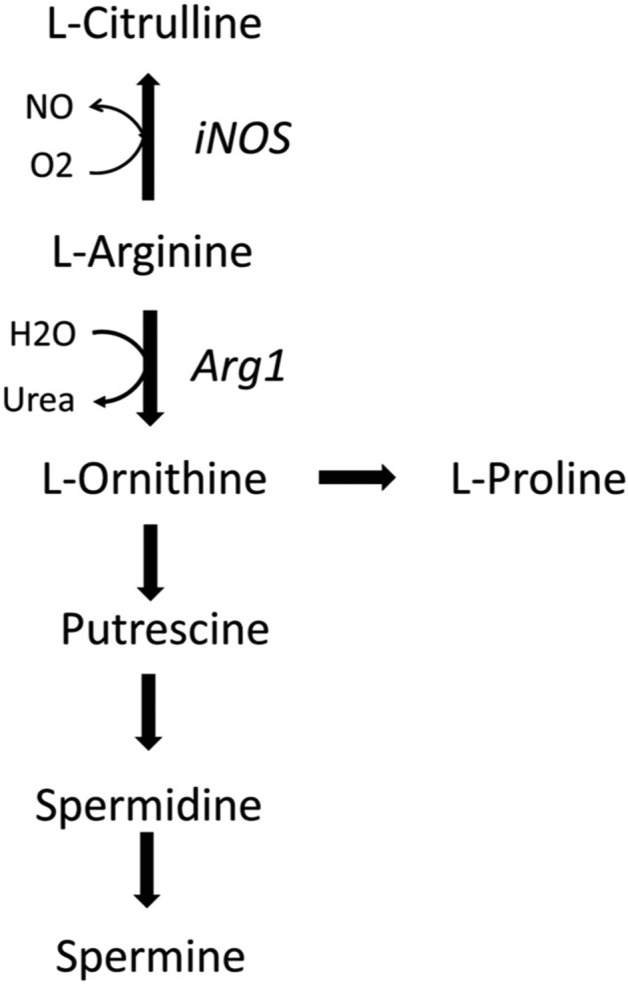
Arg catabolism. Arg breakdown is performed by iNOS and Arg1. iNOS catalyses the conversion of Arg into NO and citrulline, while Arg1 catalyses the conversion of Arg into ornithine and urea.

The relative expression of the two Arg catabolizing enzymes iNOS and Arg1, and the production of associated Arg-derived metabolites, are related to the intrinsic regulation of macrophage polarisation, function, and activation state, and to the profile and time course of the immune response against viruses and bacteria pathogens (Fig. 1) [18]. In addition to Arg, other amino acids have been involved in macrophages polarisation, such as Gln or serine, for their role into TCA cycle and one-carbon metabolism, respectively [3].

Arg is the substrate for iNOS-mediated production of NO involved in the initial innate inflammatory immune response to viral infections [19]. In this initial phase of the innate immune responses against pathogens iNOS-expressing macrophages dominate in response to pro-inflammatory cytokines and bacterial LPS and produce NO from Arg that stimulates an inflammatory immune response and a cytotoxic and cytostatic activity to eradicate pathogens. In experimental animal models, severe influenza virus infection induces NO overproduction and pulmonary hyperinflammation causally related to morbidity [20]. Severe cases of COVID-19 are also characterised by NO overproduction, lung hyperinflammation and local tissue damage [21]. Arg depletion using Arg-depleting enzymes represents a promising therapeutic antiviral approach in patients with SARSCoV-2 infection. Reduction of plasma Arg levels could limit the production of NO by iNOS and the extent of the hyperinflammatory response, as previously observed with ADI-PEG20 in patients with hepatocellular carcinoma and hepatitis C virus infection [22]. Several developed arginine-metabolising enzymes may lower plasma Arg and NO-dependant hyperinflammatory response in COVID-19 and other viral diseases [23].

Moreover, Arg depletion may directly impact SARSCoV-2 lifecycle. Arg appears a key amino acid for viral replication of many DNA and RNA viruses and several steps in the SARSCoV-2 replication rely on viral proteins with conserved Arg residues. Arg depletion may inhibit SARS-CoV-2 replication and packing as previously observed for other virus of the families Herpesviridae and Adenoviridae [23, 24]. In vitro data showed that the Arg-depleting enzyme pegzilarginase inhibits SARS-CoV-2 replication in Vero cells [23]. Additionally, Arg residues at position 407 and at the S1/S2 site of the spike protein of SARS-CoV-2 could be involved in the binding of the virus to the host cell receptor angiotensin-converting enzyme 2 and in the fusion of the viral membrane with the host cell membrane [25–27].

In the second phase of the immune response to pathogen infection, Arg1-expressing macrophages dominate in response to Th2-type cytokines and provide Pro and polyamines that participate to modulate the hyperinflammation and to tissue repair [18]. However, viruses and hosts compete for polyamines that are involved in host tissue repair but are also critical for the virus genome packaging and viral enzymatic activity. Thus, any pharmacological antiviral strategy targeting Arg and polyamines production should favour the host while restricting the use of polyamines for virus replication [28].

## Tryptophan and the tryptophan-catabolizing enzyme IDO1

**Fig. 2:**
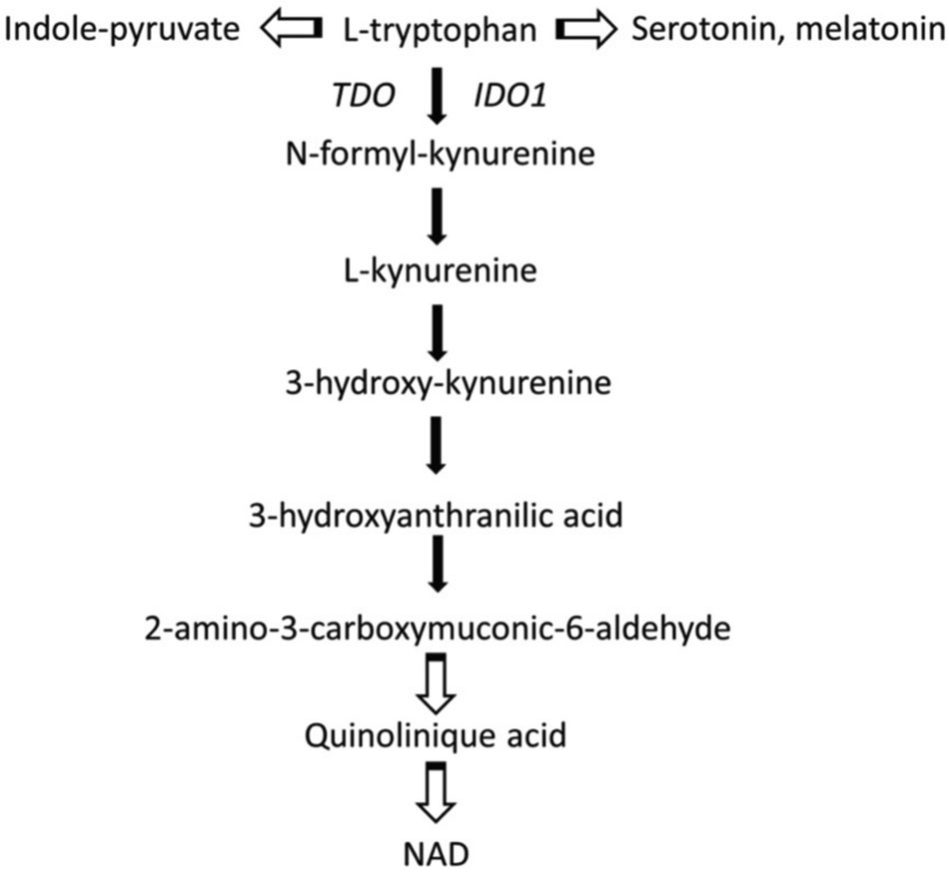
Tryptophan catabolism and the kynurenine (Kyn) pathway. In mammals, the Kyn pathway is the major route for Trp catabolism. Alternatively, Trp can be converted by tryptophan hydroxylase-1 to 5-hydroxytryptophan, a precursor in the pathway for the synthesis of serotonin and melatonin.

The essential amino acid tryptophan (Trp) is a precursor for the synthesis of proteins and for several molecules involved in the regulation of diverse biological processes, including the amino acid kynurenine (Kyn), NAD, serotonin and melatonin [29]. In mammals, the Kyn pathway is the major route for Trp catabolism while a lower amount is catabolised via the methoxyindole pathway. The conversion of Trp into Kyn involves the two enzymes, indoleamine-2,3- dioxygenase 1 (IDO1) and tryptophan-2,3-dioxygenase (TDO) (Fig. 2). IDO1 is a monomeric, haem-containing enzyme that catalyses the initial, rate-limiting step in the degradation of Trp into L-Kyn [30, 31]. Alternatively, Trp can be converted by tryptophan hydroxylase-1 to 5-hydroxytryptophan, a precursor in the pathway for the synthesis of serotonin and melatonin [32].

The enzyme IDO1, mainly expressed by DCs, is involved in the control of acute inflammation by an immunoregulatory immunosuppressive effects linked to its catabolic activity that induce a rapid change of Trp and Kyn levels that causes Trp deprivation in the microenvironment and the generation of immunoactive Kyn metabolites [33]. Kyn is an endogenous ligand for aryl hydrocarbon receptor (AhR) that affects the biology of immune cells and cancer cells [34]. The Kyn-derived catabolites such as quinolinic acid and 3-hydroxyanthranilic acid have different biological activities, acting as immunosuppressive factors and inhibiting T-cell proliferation [35, 36]. The cytokine γ-IFN activates intense short-term IDO1-competent DCs mediated immunosuppressive activity on T lymphocytes, including inhibition of proliferation, apoptosis, and differentiation towards a regulatory phenotype Treg [37]. A main effect of a defective IDO1 catalytic activity is the low generation of Kyn-type ligands of the AhR and of Treg cells [37, 38]. A defective activity of IDO1 was previously shown in autoimmune and chronic inflammatory diseases [39, 40].

Identification of mechanisms that control the functions and fate of IDO1 opens new strategy for the pharmacologic control of IDO1 functioning and its immunoregulatory effect and modulating IDO1 catalytic activity is a promising therapeutic approach to modulate the immune response during the different steps of the immune response to pathogens infection. For some viral infections high IDO1 activity represents a main cause of immune unresponsiveness during the initial immune responses that aims to eradicate pathogens and downregulation of the short-term immunosuppressive regulatory effects of IDO1 should improve this short-term immune response. In contrast, during the second phase of the response, upregulating and increasing the activity of IDO1 could participate to prevent harmful hyperinflammation.

At the crossroad between Arg and Trp metabolism and their regulatory effects, the two enzymes Arg1 and IDO1 are involved in the secondary control of acute hyperinflammation associated to pathogens infection by an immunoregulatory immunosuppressive effects via either amino acid depletion (as for Arg1) or via the combined effects of immunoregulatory Kyn and signalling activity (as for IDO1) [3, 32]. However, these two pathways may have separate functions according to different environment conditions and could be complementary associated in therapeutic approaches. Interestingly, an IL4-dominated cellular environments promote Arg1 expression (mainly in macrophages/MDSCs) [14], while an γ-IFN-dominated cellular environments promote IDO1 (DCs) expression [41]. The interaction between Arg and Trp metabolism in the immunoregulatory processes is also illustrated by the observation that spermidine activates molecular pathways related to the activation of IDO1 and confers to DCs a tolerogenic phenotype [42].

## Glutamine and host defence

Rapidly proliferating cells, including several immune cells, require a supply of some amino acids such as Gln and upregulate metabolic pathways providing these amino acids, and these processes can be used as therapeutic targets against infection [43]. The amino acid Gln is a non-essential amino acid that can become conditionally essential during periods of physiological stress or under critical illness and injury [44]. Gln is essential for proliferating cells, including lymphocytes, thymocytes, and colonocytes, where it is actively used in several important metabolic processes. Gln is involved in the synthesis of protein, is the precursor for nucleotides and amino sugars, is degraded by glutaminase to Glu that is further metabolised to different products including g-amino butyrate, glutathione, and folic acid, and is a main source of energy by providing intermediates components of the tricarboxylic acid cycle. Gln is the most abundant free amino acid in human muscles and plasma and is utilised at high rates by rapidly dividing cells, including leucocytes, to provide energy and for nucleotide biosynthesis. The expression of several genes in immune system cells is largely dependent on Gln availability.

The degradation of Gln leads to the formation of NH_3_ and aspartate and to the synthesis of the nucleotides purines and pyrimidines involved in the synthesis of DNA and RNA. Gln acts as a nitrogen donor for rapidly dividing cells, such as lymphocytes, in which it is critical for nucleotide synthesis and energy production [45] and this critically impacts the efficiency of immune cell responses [46]. Glutaminases catalyse the hydrolytic deamidation of Gln to Glu and NH_3_ and in mammals glutaminase is highly expressed in lymphocytes and macrophages of lymphoid organs and increase in response to inflammatory stimuli [47]. Gln acts as an energy substrate for leucocytes and plays an essential role in cell proliferation, tissue repair process activity, and intracellular pathways associated with pathogen recognition [48]. The TCA cycle of M2 macrophages has no metabolic pathway for substrate flux deviation while M1 macrophages (treated with LPS) have two points of substrate flux deviation, one occurring at the isocitrate dehydrogenase step reaction and another one at post succinate formation. This results in an accumulation of TCA cycle intermediates (e.g., succinate, a-ketoglutarate, citrate, and itaconate) that regulates LPS macrophage activation. Itaconate has anti-inflammatory properties through activation of nuclear factor erythroid 2-related factor 2 via Kelch-like ECH-associated protein 1 alkylation.

Naive T lymphocytes are rapidly activated following pathogens invasion and Gln is essential for their proliferation and biosynthetic activities requiring high energy supply met by increased absorption and use of Gln [49–51]. During the activation of T lymphocytes Gln transporters including ASCT2 are upregulated inducing a rapid Gln uptake while a reduced activity of these transporters impairs the function of these immune cells [50, 52, 53]. Gln is involved in the control of proliferation of immune system cells through activation of proteins such as ERK and JNK kinases. Both proteins act on the activation of transcription factors such as JNK and AP-1, leading to the transcription of cell proliferation-related genes. Appropriate Gln concentration leads to the expression of key lymphocyte cells surface markers, such as CD25, CD45RO, and CD71, and the production of cytokines, such as IFN-g, TNF-a, and IL-6. Gln promotes the proliferation of activated human T lymphocytes and stimulated PBMCs, the production and the secretion by these cells of‑γ-IFN and IL‑2, and Gln depletion inhibits T lymphocyte proliferation, and decreases IL‑2 and IFN‑γ production [50, 54, 55]. In addition to its role as a fuel, Gln regulates immuno-inflammatory responses, and antioxidant status. Administration of Gln regulates the inflammation derived by gut starvation [56]. Gln and other amino acids may also exert their action via redox regulation in immune cells, which modulates many cellular functions. Gln (via glutamate), cysteine, and glycine are the precursor amino acids for the synthesis of GSH. However, among these three amino acids, glutamate represents the first and probably the most important limiting step in the synthesis of GSH intermediate compounds [57]. Glutamate synthesis, in turn, is dependent on the Gln intracellular availability. Thus, a higher Gln/glutamate ratio reinforces the substrate availability for GSH synthesis.

Gln depletion alters the balance of the immune response that become more suppressive, by favouring a regulatory T phenotype rather than a Th1 phenotype, even in a cytokine environment inducing the Th1 phenotype, associated with an inhibition of IFN‑γ secretion [49]. Gln complementation showed antiviral activity against the herpes virus [58, 59] and a complement associating zinc, vitamin D and Gln displayed a potential adjuvant and synergistic therapeutic potential in modulating antiviral immune defences functions through γ-IFN signalling in the treatment of COVID‑19 [60]. However, as virus-infected host cells have an increase in Gln utilisation, development of antiviral strategy targeting Gln must deplete Gln in the environment of virus-infected host cells while favouring Gln utilisation by host immune cell system [61].

## Other amino acid metabolism and signalling pathways

Amino acid sufficiency and deficiency and amino acid and protein metabolism are controlled by the amino acid sensing pathways mTOR and GCN2 with a crosstalk between the two pathways [62]. In response to amino acid abundance mTOR activates the host protein translation and coronaviruses exploit this cellular machinery for their own protein synthesis and replication [63]. The high risk of people with obesity to harmful hyperinflammatory morbidity with SARS-CoV-2 infection could be for a part associated to the excess amino acid and mTOR hyperactivation making a favouring environment for coronaviruses including SARS-CoV-2 that use the host translational machinery for viral replication. In contrast, it was observed that in response to amino acid starvation the amino acid sensor GCN2 activates downstream pathways that restrict inflammation and viral replication [64–66]. Activation of GCN2 was shown to restrict viral invasion during viral infection such as dengue virus infection, with upregulation of autophagy, inhibition of pro-inflammatory IL-1β cytokine and intestinal inflammation, and decreased hyperinflammation and cytokine storm [64–66]. In this context, using bioactive pharmacological molecules to activate the amino acid starvation sensor GCN2 could participate to reduce both virus infection and the hyperinflammation in patients with SARS-CoV-2 infection [66].

Another example relies to serine (Ser) that is also required in large amount for rapidly growing cells [67–69]. Ser is supplied to cells through uptake from the extracellular environment, and de novo produced by transamination via glycine or from glucose and Glu [70]. Ser is taken up across cell membranes through either the sodium-independent neutral amino acid exchanger (SLC7A10/SLC3A2) mainly expressed in the nerve cell membrane, or the sodium-dependent neutral amino acid exchangers SLC1A4/SLC1A5 expressed in other tissues. Proliferating activated immune cells are highly dependent on Ser, activation and differentiation of T cells need enough Ser and dietary Ser restriction can inhibit their proliferation [68, 71]. SLC1A5 deficiency impairs the differentiation of T helper 1 (Th1) and Th17 cells and attenuate inflammatory responses by inhibiting T cell receptor-activated mTORC1 [52]. Ser can be de novo synthesised from glycine transamination by the serine hydroxymethyltransferase (SHMT1/2) or produced from 3-phosphoglycerate (3-PG), an intermediate of glycolysis [72]. Ser directly modulates adaptive immunity by controlling T cell proliferative capacity by supplying one-carbon units for de novo nucleotide biosynthesis in proliferating T cells [71] and for the generation of SAM to maintain a high SAM:S-adenosyl-homocysteine ratio to support the epigenetic methylation of histone and the production of some immune cytokines, including IL-1β. SAM appears essential for inflammatory macrophages and reducing SAM generation has an anti-inflammatory effect [73, 74]. Interestingly, Epstein–Barr virus infection induces a control of NADPH levels in infected cells by maintaining intracellular Ser level to augment one-carbon flux for B cell proliferation [75].

In the same way, the two sulphur-containing amino acids, the essential amino acid methionine (Met) and the semi-essential amino cysteine (Cys), are also involved in immunoregulation. Cys is strictly required for T-cell proliferation because T cells lack the enzyme converting Met to Cys and must import Cys via their transport system [76]. Cys availability is therefore critical for T-cell functions, with cells either providing (as is the case for antigen-presenting cells) or sequestering (in particular, MDSCs) this amino acid, resulting in stimulatory or suppressive effects, respectively. Cys dioxygenase (CDO) catabolises Cys to cysteine sulfinic acid, and further to hypotaurine and to taurine, or to pyruvate and sulfite. Thus, CDO not only removes Cys cytotoxic excess, but is also necessary to produce hypotaurine/taurine and sulfite/ sulfate from Cys [77]. The combination of copper, N-acetylcysteine, colchicine, and NO associated to currently used experimental antiviral agents, has been proposed as a potential treatment in SARSCOV-2-infected patients [78]. Moreover, the coronavirus RNA cap structure is methylated by two viral methyltransferases that transfer methyl groups provided by S-adenosylmethionine (SAM). The proper methylation of the virus depends on the level of Met availability in the host to form SAM and it has been proposed to restrict Met availability by treating Covid-19 patient with oral recombinant methioninase [79]. Restricting Met not only inhibits viral replication, which depends on the viral RNA cap methylation, but also inhibits the proliferation of the infected cells, which have an increased requirement for Met. Most importantly, the virally induced T-cell- and macrophage-mediated cytokine storm, a significant cause of morbidity, can also be inhibited by Met depletion, since T-cell and macrophages activation requires a supply of Met.

Moreover, the interleukin 4-inducible 1 enzyme, secreted or localised in lysosomes, catalyses Phe catabolism leading Phe depletion and to the production of the a-ketoacid phenylpyruvate, and H2O2 with antibacterial toxic effects potentiated by the associated production of NH3 and basification of the medium [76, 80]. These processes result in the inhibition of microbial bacterial growth and to an immunosuppressive activity towards T lymphocytes [81].

## Conclusion

Amino acid metabolism and signalling processes participate to the control of pathogen infection and to the regulation of the inflammation induced by activation of innate, adaptive, and regulatory immune responses. The crosstalk between catabolism of amino acids and the immune system represents an important process for tuning immune reactivity and reducing hyperinflammation associated to immune responses to infections. Among amino acids, Arg availability and the catabolic enzyme iNOS are involved in the inflammatory innate response through the production of NO, while Arg and Trp availability, associated to the two catabolic enzymes Arg1 and IDO1, represent catabolic pathways involved in modulating immune reactivity and exerting regulatory effect on inflammation and on the adaptive immune responses. Moreover, the amino acids Gln, Ser, Cys, Met, and Phe are also being considered as amino acids involved in pathogen development and in the balance of the immune reactivity. Overall, excess amino acid and mTOR hyperactivation appear as an environment favouring virus invasion while amino acid starvation and GCN2 activation restrict viral invasion. These different mechanisms represent potential therapeutic targets to control infection and associated hyperinflammatory response causally related to morbidity and mortality induced by pathogens, including coronavirus.
