# Correction: Amino acid metabolism and signalling pathways: potential targets in the control of infection and immunity

**DOI:** 10.1038/s41387-021-00167-y

**Published:** 2021-07-08

**Authors:** Daniel Tomé

**Affiliations:** UMR PNCA, AgroParisTech, INRAE, Université Paris-Saclay, Paris, France

Correction to: *Nutrition and Diabetes*

10.1038/s41387-021-00164-1, published online 23 June 2021

The original version of this article unfortunately contained a mistake in the Abstract. The sentence “The two Arg-catabolising enzymes Arg1 and IDO1 reduce the hyperinflammation by an immunosuppressive effect via either Arg starvation (for Arg1) or via the immunoregulatory activity of the Arg-derived metabolites Kyn (for IDO1).” should read: “The two Arg- and Trp-catabolising enzymes Arg1 and IDO1 reduce the hyperinflammation by an immunosuppressive effect via either Arg starvation (for Arg1) or via the immunoregulatory activity of the Trp-derived metabolites Kyn (for IDO1).” The author apologizes for the mistake. The original article has been corrected.

